# Infections with highly pathogenic avian influenza A virus (HPAIV) H5N8 in harbor seals at the German North Sea coast, 2021

**DOI:** 10.1080/22221751.2022.2043726

**Published:** 2022-03-01

**Authors:** Alexander Postel, Jacqueline King, Franziska K. Kaiser, Johanna Kennedy, Mara Sophie Lombardo, Wencke Reineking, Madeleine de le Roi, Timm Harder, Anne Pohlmann, Thomas Gerlach, Guus Rimmelzwaan, Simon Rohner, Lotte C. Striewe, Stephanie Gross, Luca A. Schick, Jana C. Klink, Katharina Kramer, Albert D. M. E. Osterhaus, Martin Beer, Wolfgang Baumgärtner, Ursula Siebert, Paul Becher

**Affiliations:** a Institute of Virology, University of Veterinary Medicine, Hannover, Germany; b Institute of Diagnostic Virology, Friedrich-Loeffler-Institute, Greifswald, Germany; c Research Center for Emerging Infections and Zoonoses, University of Veterinary Medicine, Hannover, Germany; d Institute of Pathology, University of Veterinary Medicine, Hannover, Germany; e Institute for Terrestrial and Aquatic Wildlife Research, University of Veterinary Medicine, Hannover, Germany; f Landeslabor Schleswig- Holstein, Neumünster, Germany

**Keywords:** Influenza virus, HPAIV, H5N8, avian, seal, mammal, brain, lung

## Abstract

In brain tissue of three harbor seals of the German North Sea coast, high virus loads of highly pathogenic avian influenza virus (HPAIV) H5N8 were detected. Identification of different virus variants indicates high exposure to HPAIV circulating in wild birds, but there is no evidence for H5 specific antibodies in healthy seals. Replication of avian viruses in seals may allow HPAIV to acquire mutations needed to adapt to mammalian hosts as shown by PB2 627K variants detected in these cases.

The 2020/2021 epidemic with 3555 reported highly pathogenic avian influenza virus (HPAIV) infections in wild birds and approx. 22,400,000 affected poultry in 28 European countries appears the most devastating HPAI epidemic that ever occurred in Europe [1]. Here, we investigated unusual influenza A virus (IAV) infections detected in three dead harbor seals.

## Screening for viral pathogens in seals

Mid-August 2021, tracheal swabs and tissue samples obtained from three adult harbor seals (*Phoca vitulina*) found at the German North Sea coast were investigated for the presence of morbilliviruses, herpesviruses, and IAV [2–4] as part of the regular health monitoring performed since the seal-die-off in 1989. Two seals were found on the island of Sylt, while the third individual was found ∼130 km further south in the Meldorfer Bucht; one animal (Sylt-1) was lethargic with no signs of reactions and died while being found, the other two seals were found dead. All three harbor seals belong to the same population including 8,849 counted individuals in the Wadden Sea of Schleswig-Holstein in 2021 [5]. The seals were collected and investigated because it is uncommon for the time of the year to find dead adult harbor seals. In pooled organ samples (Meldorf-1, Sylt-1: lung, brain, intestine, spleen, kidney, liver, tonsils, mesenteric lymph node; Sylt-2: lung, brain) high IAV genome loads were detected (Meldorf-1: Cq 17; Sylt-1: Cq 24; Sylt-2: Cq 21). IAV was isolated from seals Meldorf-1 and Sylt-1 in MDCK cells under BSL3 conditions. Repeated isolation attempts in cell culture as well as in embryonated eggs remained unsuccessful for seal Sylt-2. The pooled sample as well as the individually tested brain sample from seal Sylt-1 tested also positive for herpesvirus genomes. Molecular cloning of the PCR amplicons obtained from the brain sample and subsequent sequencing revealed the presence of two genetically very distinct herpesvirus genomes. One sequence is almost (99.5%) identical to a sequence obtained from a harbor seal (GenBank NC043062) and belonging to phocine herpesvirus 2 (PhHV-2), the other is most similar (approx. 75% nucleotide identity) to a harp seal herpesvirus sequence (GenBank NC055139) assigned to PhHV-3. Tissue pools were negative for morbillivirus genomes. Interestingly, tracheal swabs of the respective seals were negative for IAV genomes and lung tissues revealed considerably lower viral genome loads compared to brain tissue: Cq 38 vs. 18 (Meldorf-1), Cq 30 vs. 22 (Sylt-1), and Cq 38 vs. 21 (Sylt-2), respectively. During a previous IAV (H10N7) outbreak in seals associated with pneumonia and slightly increased mortality [6], and in context of HPAIV H5N8 infection in lung tissues of two dead grey seals (*Halichoerus grypus*) found at the Polish Baltic coast in 2016 [4], no evidence for an infection of the brain was found. Currently circulating HPAIV H5N8 has been very recently reported to cause unusual neurological infection with fatal outcome in four harbor seals and one grey seal kept in a wildlife rehabilitation centre [7].

## Genetic characterization of the influenza viruses

**Figure 1.**
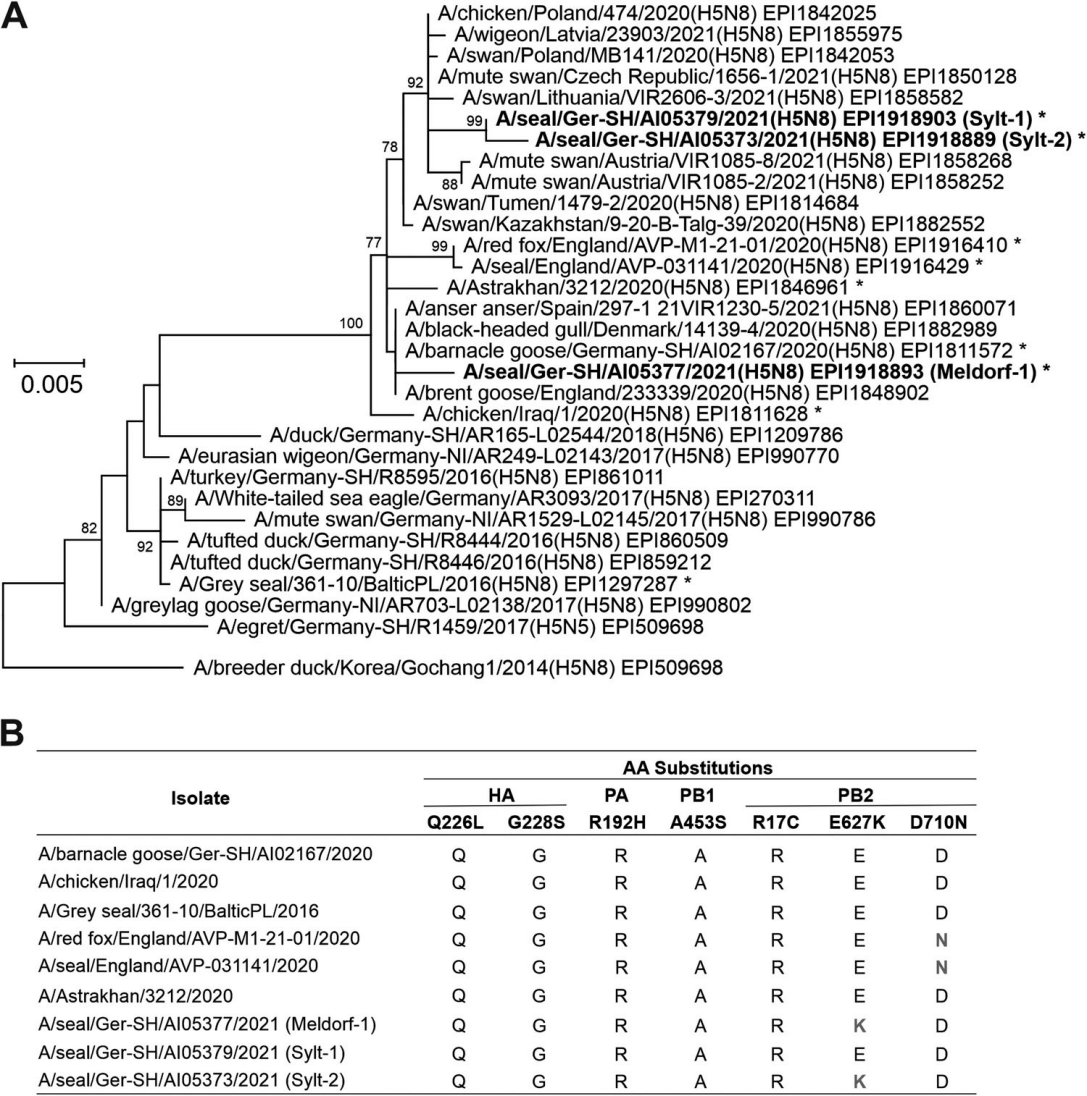
Phylogenetic analysis of hemagglutinin (HA) encoding sequences of highly pathogenic avian influenza viruses (HPAIV) subtype H5N8. (A) Complete H5 encoding nucleotide sequences obtained from brain tissues of three seals determined within this study (indicated in bold) were compared to sequences of H5N8 HPAIV previously detected in seals from Poland (2016), England (2020) and a human case (2020) from Russia [4,7,14]. In addition, a representative set of H5 sequences affiliated to clade 2.3.4.4b is included. Sequences were obtained from the EpiFlu^TM^ GISAID database and accession numbers are indicated next to the cryptograms [15]. Phylogeny was performed using a maximum likelihood approach implemented in MegaX (best fit model: HKY + G). Numbers at nodes represent bootstrap values of 70 or higher. Scale bar indicates nucleotide substitutions per site. (B) Variant amino acids in the HA, PA, PB1 and PB2 proteins are shown for selected sequences (indicated by * in the phylogenetic tree, panel A), respectively. Positions of amino acid substitutions implicated in adaptation to mammalian hosts are given in the top row.

For initial genetic characterization of the IAV, complete hemagglutinin and neuraminidase encoding sequences were established by conventional Sanger sequencing using primers as listed in Supplementary Table S1. Sequences used for sequence comparison and phylogenetic analyses were obtained from the EpiFlu^TM^ GISAID database (Supplementary Table S2). Identified subtype H5N8 strains are belonging to the goose/Guangdong lineage, clade 2.3.4.4b (Figure 1(A)). Different reassortant viruses of this clade have circulated in wild birds and poultry holdings in Europe since 2015 [8,9]. Detection of polybasic cleavage sites PLRE(K/R)RRKR/GLF within the hemagglutinin (HA) sequences of the three virus strains allowed the classification as HPAIV [10]. To investigate whether mutations implicated in adaptation to mammalian hosts occurred in the viral genomes, brain tissue samples underwent full-genome sequencing, conducted as previously described with prior amplification and nanopore sequencing methods [11]. Consensus sequences have been deposited in the EpiFlu^TM^ GISAID database under accession numbers EPI_ISL_4805852 (Meldorf-1), EPI_ISL_4805936 (Sylt-1), and EPI_ISL_4804850 (Sylt-2).

Distinct NP segments allowed to distinguish two genotypes. Virus from seal Meldorf-1 is similar to the HPAIV H5N8 genotype Ger-10-20-N8 that dominated the current avian epizootic 2020/2021 [8], and was found in Germany from October 2020 until July 2021. In comparison, viruses originating from seals found on Sylt (Sylt-1 and Sylt-2), clustered with genotype Ger-02-21-N8, a much rarer genotype with a novel NP segment sharing the highest identity with Eurasian LPAIV strains found in wild birds and poultry holdings during the past years. HPAIV genotype Ger-02-21-N8 was identified so far only in three poultry holdings in Germany in February and March 2021, but has been most likely also circulating undetected in wild birds. Both harbor seal isolates from Sylt share 99.82% identity between all concatenated segments, while they showed <98.6% identity to the isolate from seal Meldorf-1. Accordingly, at least two independent entries into the seal population are very likely (Supplementary Figure S1).

Screening for mutations indicating a putative avian to mammalian host shift showed the presence of the previously described E627K mutation in the PB2 segment [12] in the consensus sequences of Meldorf-1 and Sylt-2 (Figure 1(B)). A minor variant analysis of the next-generation sequencing data from two independently sequenced samples of Sylt-2 at this position showed a mixture of 59.2% mammalian (627K) with 40.8% avian (E627) variant. Meldorf-1 carried a dominant 627K variant (97.9%) with virtually no traces of the avian-adapted amino acids (AA) left. In contrast, Sylt-1 showed only the avian variant (E627). Albeit further in-depth analysis of the sequencing data, no other detections of known and previously described host shift AA sites could be detected in any sample (Figure 1(B)). The detected E627K mutation in PB2 could potentially demonstrate one step involved in the adaptation of avian HPAIV to a mammalian host and indicates the potential role of seals implicated in host shifts.

## Currently circulating HPAIV (H5N8) can cause infections of the central nervous system of seals

Histopathological investigations were performed to characterize HPAIV infection in the brain and lung tissues (Supplementary Table S3, Supplementary Figure S2A). Seal Meldorf-1 showed a mild to moderate, lymphohistiocytic meningoencephalitis with few neutrophils and hemorrhage (Supplementary Figure S2A). Hematoxylin and eosin stained slides were evaluated semiquantitatively for the mean number of infiltrating inflammatory cells (for classification see Supplementary Material Table S2, Figure S2). Additional lesions included moderate, multifocal, lymphohistiocytic vasculitis and single cell necrosis in the surrounding tissue affecting mainly glial cells and eosinophilic, shrunken, triangular, necrotic neurons as well as fading neurons within cerebrum and cerebellum (Supplementary Figure S2B). Furthermore, severe, diffuse, acute hyperemia of the lung and an alveolar emphysema and oedema was detected in this animal. Similar alterations were found in brain and lung of the two other seals (Supplementary Table S3). Although seal Sylt-1 tested positive for two different herpesvirus genomes, typical histopathological lesions, such as intranuclear inclusion bodies, were not detected. Due to the lack of PhHV-2 and PhHV-3 specific antibodies, it was not possible to evaluate the contribution of herpesviruses to the brain lesions of Sylt-1 by immunohistochemistry. Additionally, the lung of seal Sylt-2 showed mild to moderate multifocal lymphohistiocytic infiltrates with single cell necrosis and few nematode larvae. Immunohistochemistry revealed high (Meldorf-1, Supplementary Figure S2C) and low (Sylt-1) numbers of IAV nucleoprotein positive cells in the brain tissue, respectively. Nucleoprotein was detected in neurons, glial cells and in the neuropil accentuated within the inflammatory lesions (Supplementary Figure S2D). No IAV antigen was detectable within the brain of seal Sylt-2 and the lungs of all three animals. The results of these investigations correlate with the differences in viral genome loads of lung and brain tissues detected by RT-qPCR. The lack of IAV nucleoprotein detection in the brain of animal Sylt-2 could be due to the poor preservation status of the investigated tissue.

## No evidence for exposure of living seals to circulating HPAIV H5

Twenty clinically healthy harbor seals were temporarily captured at the Lorenzensplate (located between the island of Sylt and the Meldorfer Bucht) end of August 2021 for the yearly health monitoring which included sampling of nasal swabs and serum. None of the swab samples contained detectable IAV genomes. Moreover, all sera from these 20 animals and 25 additional sera from juvenile Dutch harbor seals collected during three months prior to the seal deaths were tested negative for H5 specific antibodies in ELISA (ID Screen; 1:2 dilutions) as well as in a virus neutralization assay using the H5N8 isolate obtained from seal Meldorf-1, respectively.

## Discussion and conclusions

This study describes unusual infections of three adult harbor seals from the German Wadden Sea with HPAIV H5N8 of clade 2.3.4.4b. Most closely related viruses circulate in wild water birds around the North Sea since fall 2020. Serological investigations did not provide evidence for a wide distribution of these viruses in healthy seals. Similar to very recently reported findings in captive seals in the UK [7], our results show that HPAIV H5N8 of clade 2.3.4.4b can induce fatal CNS infections in seals under natural conditions. Experimental infection of cats has elucidated that HPAIV reaches the brain through the gut, *Plexus Auerbachii*, and *Nervus vagus* [13]. Thus, the most probable route of infection of the seals is oral uptake of contaminated water, faeces or infected birds. The highest viral genome loads were present in brain tissues, while in lung tissue and tracheal swabs only low or no viral loads were detectable. Therefore, passive surveillance for HPAIV in seals and other mammalian species should not be limited to swabs and lung samples but should also include brain tissue. There was no evidence that histological alterations in the brain of one seal (Sylt-1) are primarily caused by infection with PhHV, although a contribution cannot be fully excluded. Two HPAIV isolated from the German seals contain the PB2 E627K mutation implicated in adaptation to mammalian hosts. The genetic findings underline the role of seals as a putative “adaptive vessel” for avian influenza viruses and the importance of surveillance in wild bird and mammal populations.

## Supplementary Material

Supplemental Material

## References

[1] EFSA (European Food Safety Authority), EECfD, Prevention and Control, EERLfAI, et al. Scientific report: avian influenza overview February–May 2021. 2021.

[2] VanDevanter DR, Warrener P, Bennett L, et al. Detection and analysis of diverse herpesviral species by consensus primer PCR. J Clin Microbiol. 1996 Jul;34(7):1666–1671. 8784566 10.1128/jcm.34.7.1666-1671.1996 PMC229091

[3] Verna F, Giorda F, Miceli I, et al. Detection of morbillivirus infection by RT-PCR RFLP analysis in cetaceans and carnivores. J Virol Methods. 2017 Sep;247:22–27. 28528278 10.1016/j.jviromet.2017.05.009

[4] Shin DL, Siebert U, Lakemeyer J, et al. Highly pathogenic avian influenza A (H5N8) virus in gray seals, Baltic Sea. Emerg Infect Dis. 2019 Dec;25(12):2295–2298. 31742519 10.3201/eid2512.181472 PMC6874272

[5] Galatius A, Abel C, Brackmann J, et al. EG-marine mammals harbour seal surveys in the Wadden Sea and Helgoland 2021. Common Wadden Sea Secretariat; 2021.

[6] Bodewes R, Bestebroer TM, van der Vries E, et al. Avian influenza A(H10N7) virus-associated mass deaths among harbor seals. Emerg Infect Dis. 2015 Apr;21(4):720–722. 25811303 10.3201/eid2104.141675 PMC4378483

[7] Floyd T, Banyard AC, Lean FZX, et al. Encephalitis and death in wild mammals at a rehabilitation center after infection with highly pathogenic avian influenza A (H5N8) virus, United Kingdom. Emerg Infect Dis. 2021 Nov;27(11):2856–2863. 34670647 10.3201/eid2711.211225 PMC8544989

[8] Lewis NS, Banyard AC, Whittard E, et al. Emergence and spread of novel H5N8, H5N5 and H5N1 clade 2.3.4.4 highly pathogenic avian influenza in 2020. Emerg Microbes Infect. 2021 Dec;10(1):148–151. 33400615 10.1080/22221751.2021.1872355 PMC7832535

[9] Lycett SJ, Pohlmann A, Staubach C, et al. Genesis and spread of multiple reassortants during the 2016/2017 H5 avian influenza epidemic in Eurasia. Proc Natl Acad Sci USA. 2020 Aug 25;117(34):20814–20825. 32769208 10.1073/pnas.2001813117 PMC7456104

[10] Gall A, Hoffmann B, Harder T, et al. Universal primer set for amplification and sequencing of HA0 cleavage sites of all influenza A viruses. J Clin Microbiol. 2008 Aug;46(8):2561–2567. 18562585 10.1128/JCM.00466-08 PMC2519470

[11] King J, Harder T, Beer M, et al. Rapid multiplex MinION nanopore sequencing workflow for influenza A viruses. BMC Infect Dis. 2020 Sep 3;20(1):648. 32883215 10.1186/s12879-020-05367-y PMC7468549

[12] Subbarao EK, London W, Murphy BR. A single amino acid in the PB2 gene of influenza A virus is a determinant of host range. J Virol. 1993 Apr;67(4):1761–1764. 8445709 10.1128/jvi.67.4.1761-1764.1993 PMC240216

[13] Kuiken T, Rimmelzwaan G, van Riel D, et al. Avian H5N1 influenza in cats. Science. 2004 Oct 8;306(5694):241. 15345779 10.1126/science.1102287

[14] Pyankova OG, Susloparov IM, Moiseeva AA, et al. Isolation of clade 2.3.4.4b A (H5N8), a highly pathogenic avian influenza virus, from a worker during an outbreak on a poultry farm, Russia, December 2020. Euro Surveill. 2021 Jun;26(24):2100439. 10.2807/1560-7917.ES.2021.26.24.2100439 PMC8212591 34142650

[15] Anonymous. GISAID initiative 2008–2021. Available from: https://www.gisaid.org/.

