# Supplementary material for: Infections with highly pathogenic avian influenza A virus (HPAIV) H5N8 in harbor seals at the German North Sea coast, 2021
Source: Emerg Microbes Infect. 2022 Mar 1;11(1):725–9. doi: 10.1080/22221751.2022.2043726 (PMC8890524; doi:10.1080/22221751.2022.2043726)
Supplement: Supplemental Material [file TEMI_A_2043726_SM9735.zip › Suppl files/Suppl_Fig_S1.docx]

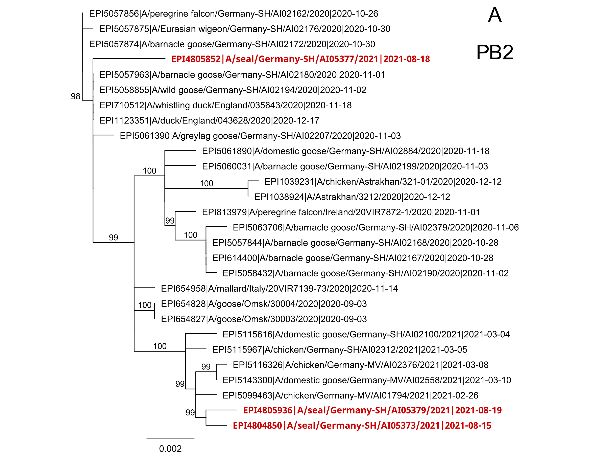


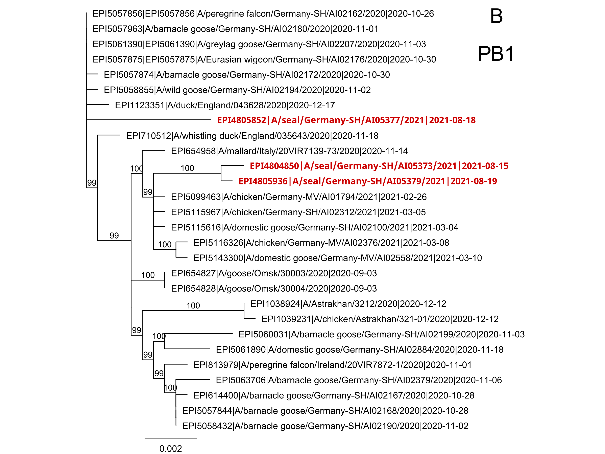


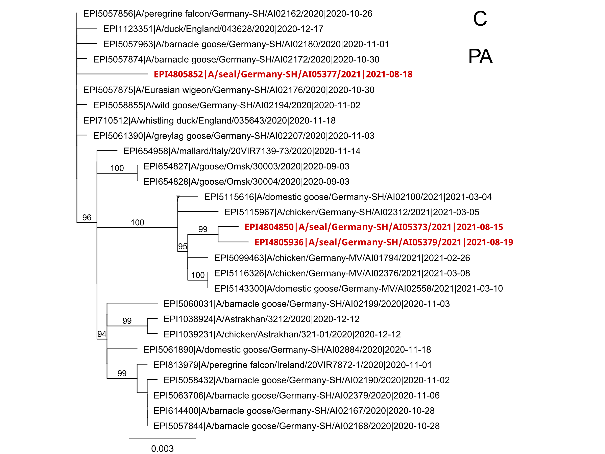


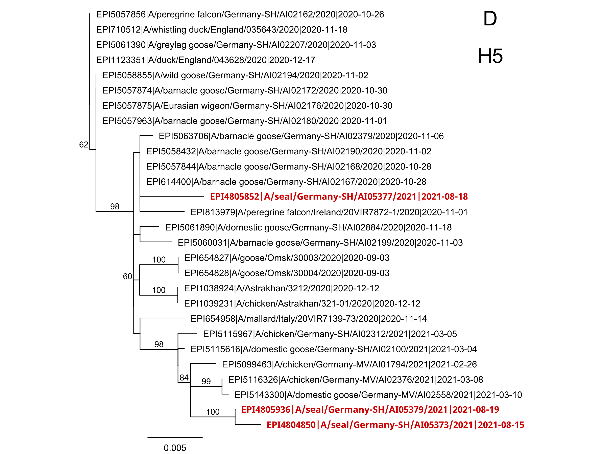


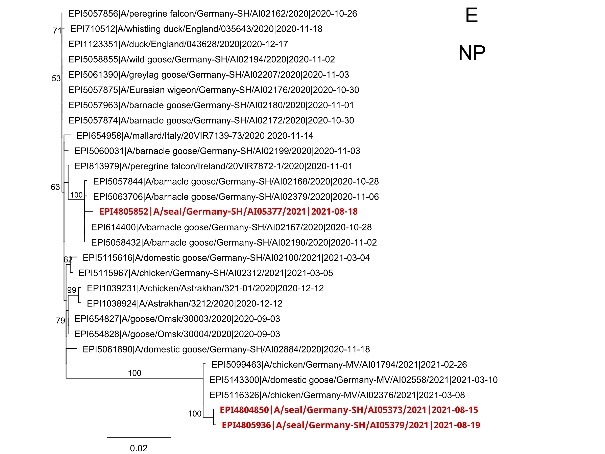


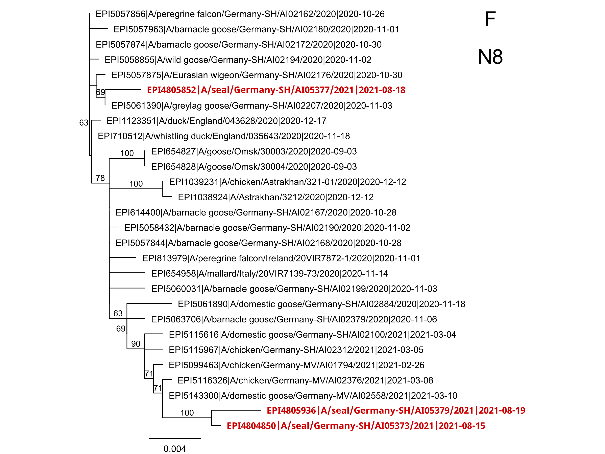


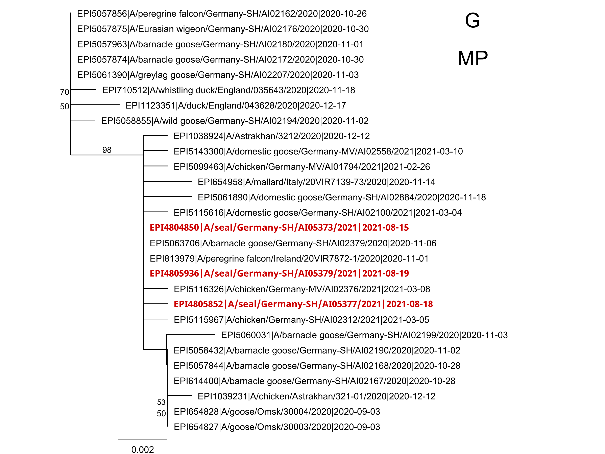


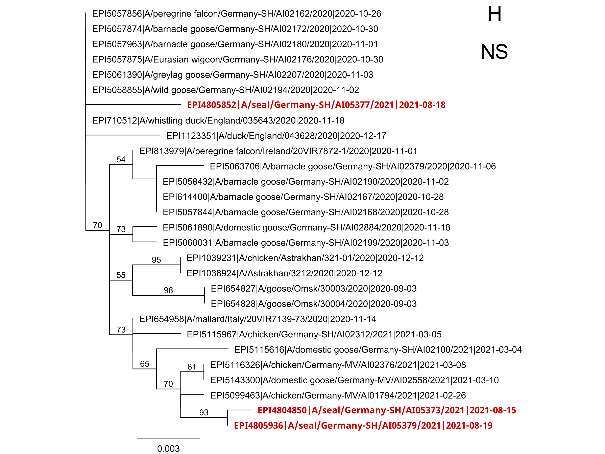


Supplementary Figure S1. Maximum likelihood trees with sequences Sylt-1, Sylt-2 and Meldorf-1 (highlighted in bold red) in addition to further available (international) HP H5N8 sequences from the 2020-2021 AIV epizootic in Europe. Trees were generated with RAxML (model GTR GAMMA with rapid bootstrapping) and search for the best scoring maximum likelihood tree (1000 bootstrap replicates). (A) PB2, (B) PB1, (C) PA, (D) H5, (E) NP, (F) N8, (G) MP, (H) NS
