# Supplementary material for: Infections with highly pathogenic avian influenza A virus (HPAIV) H5N8 in harbor seals at the German North Sea coast, 2021
Source: Emerg Microbes Infect. 2022 Mar 1;11(1):725–9. doi: 10.1080/22221751.2022.2043726 (PMC8890524; doi:10.1080/22221751.2022.2043726)
Supplement: Supplemental Material [file TEMI_A_2043726_SM9735.zip › Suppl files/Suppl-Table_S1.docx]

Supplementary Table S1. Influenza virus specific primers used in this study.

| **primer** | **sequence (5‘-3‘)** | **target** | **application** | **reference** |
| --- | --- | --- | --- | --- |
| NP-1448-F | GGGAGTCTTCGAGCTCTC | NP | real-time PCR | 1 |
| NP-1543-R | GCATTGTCTCCGAAGAAATAAGA |  |  |  |
| NP-1473-FAM | [6FAM]-AAGGCAVCGARCCCGATCGTGC-[TAM] |  |  |  |
| HA-1057.1-F | GGRGAATGCCCCAAATAYGT | HA | determination of subtype and sequence encoding cleavage site (PCR, Sequencing) | 2 |
| HA-1057.2-F | GGRARATGCCCCAGRTATGT |  |  |  |
| HA-1057.3-F | GGRGAATGCCCCAARTAYAT |  |  |  |
| HA-1232.1-R | CTGAGTCCGAACATTGAGTTGCTATGVTGRTAWCCATACCA |  |  |  |
| HA-1232.2-R | CTGAGTCCGAACATTGAGTTYTGATGYCTGAADCCRTACCA |  |  |  |
| Uni-12 | AGCAAAAGCAGG | universal | cDNA synthesis | 3 |
| H5-uni-f | CAGGGGTTCAATCTGTCAAAATGGA | H5 | PCR (H5-mid-1035r), sequencing (Meldorf-1) | 4 |
| H5-start-f | GCAGGGGTTCACTCTGTCAAAATG | H5 | PCR (H5-mid-1035r), sequencing (Sylt-1) | this study |
| H5-mid-1035r | CTATTTCTGAGCCCAGTCGCAAGG | H5 | PCR (H5-start/uni-f), sequencing | this study |
| H5-mid-815f | CCAGAAWATGCATACAAAATTGTCAARAA | H5 | PCR (H5-uni-r), sequencing | 4 |
| H5-uni-r | ACAAGGGTGTTTTTAACTACAATCTGAACTC | H5 | PCR (H5-mid-815f), sequencing | 4 |
| N8-uni-f | AGCAAAAGCAGGAGTTTAAAATGAATCCAA | N8 | PCR (N8-mid-762r), sequencing | 4 |
| N8-mid-762r | GCKGGTCCRTCHGTCATYACCCA | N8 | PCR (N8-uni-f), sequencing | 4 |
| N8-mid-597f | TGYCAYGATGGRAAGAARTGGATGA | N8 | PCR (Ba-NA-1413r), sequencing | 4 |
| Ba-NA-1413r | ATATGGTCTCGTATTAGTAGAAACAAGGAGTTTTTT | N8 | PCR (N8-mid-597f), sequencing | 3 |

1; T. Harder, National Reference Laboratory for Avian Influenza, Friedrich-Loeffler-Institute, Greifswald, Germany;

2; Gall *et al.*, 2008; doi 10.1128/JCM.00466-08;

3; Hofmann *et al*., 2001; doi 10.1007/s007050170002;

4; Shin *et al.*, 2019; doi 10.3201/eid2512.181472.
