# Supplementary material for: Infections with highly pathogenic avian influenza A virus (HPAIV) H5N8 in harbor seals at the German North Sea coast, 2021
Source: Emerg Microbes Infect. 2022 Mar 1;11(1):725–9. doi: 10.1080/22221751.2022.2043726 (PMC8890524; doi:10.1080/22221751.2022.2043726)
Supplement: Supplemental Material [file TEMI_A_2043726_SM9735.zip › Suppl files/Suppl-Table_S2_clean.docx]

Supplementary Table S2. Origin of genome sequences used for phylogenetic analyses.

| **Segment ID** | **Country** | **Collection Date** | **Isolate name** | **Originating laboratory** | **Submitting laboratory** | **Authors** |
| --- | --- | --- | --- | --- | --- | --- |
| EPI509698 | Korea | 2014-01-16 | A/breeder duck/Korea/Gochang1/2014 | - | - | Lee, Kang *et al*. |
| EPI859212 | Germany | 2016-11-07 | A/tufted duck/Ger.-SH/R8446/2016 | - | FLI | - |
| EPI860509 | Germany | 2016-11-07 | A/tufted duck/Ger.-SH/R8444/2016 | - | FLI | - |
| EPI861011 | Germany | 2016-11-09 | A/turkey/Ger.-SH/R8595/2016 | - | FLI | - |
| EPI1297287 | Poland | 2016-11-27 | A/Grey seal/361-10/BalticPL/2016 | Skóra Station | TiHo | - |
| EPI1270311 | Germany | 2016-12-29 | A/White-tailed sea eagle/Ger./AR3093/2017 | FLI | FLI | - |
| EPI990770 | Germany | 2017-01-04 | A/europ.wigeon/Ger.-NI/AR249-L02143/2017 | - | FLI | - |
| EPI990802 | Germany | 2017-01-25 | A/greylag goose/Ger.-NI/AR703-L02138/2017 | - | FLI | - |
| EPI978612 | Germany | 2017-02-14 | A/egret/Ger.-SH/ R1459/2017 | - | FLI | - |
| EPI990786 | Germany | 2017-02-14 | A/mute swan/Ger.-NI/AR1529-L02145/2017 | - | FLI | - |
| EPI1209786 | Germany | 2018-03-19 | A/duck/Ger.-SH/AR165-L02544/2018 | - | FLI | - |
| EPI1811628 | Iraq | 2020-05-12 | A/chicken/Iraq/1/2020 | CVL | APHA | - |
| EPI654828 | Russian Fed. | 2020-09-03 | A/goose/Omsk/30003/2020 | VECTOR | VECTOR | Goncharova, N., Susloparov I. *et al.* |
| EPI654827 | Russian Fed. | 2020-09-03 | A/goose/Omsk/30004/2020 | VECTOR | VECTOR | Goncharova, N., Susloparov I. *et al.* |
| EPI1814684 | Russian Fed. | 2020-09-10 | A/swan/Tumen/1479-2/2020 | ARRIAH | ARRIAH | Zinyakov, Akshalova *et al*. |
| EPI1882552 | Kazakhstan | 2020-09-21 | A/swan/Kazakhstan/9-20-B-Talg-39/2020 | NRTSV | NCB | Asylulan, Shevtsov, Karibayev *et al*. |
| EPI5057856 | Germany | 2020-10-26 | A/peregrine falcon/Germany-SH/AI02162/2020 | LSH | FLI | - |
| EPI1811572 | Germany | 2020-10-28 | A/barnacle goose/Germany-SH/AI02167/2020 | LSH | FLI | - |
| EPI614400 | Germany | 2020-10-28 | A/barnacle goose/Germany-SH/AI02167/2020 | LSH | FLI | - |
| EPI5057844 | Germany | 2020-10-28 | A/barnacle goose/Germany-SH/AI02168/2020 | LSH | FLI | - |
| EPI5057874 | Germany | 2020-10-30 | A/barnacle goose/Germany-SH/AI02172/2020 | LSH | FLI | - |
| EPI5057875 | Germany | 2020-10-30 | A/Eurasian wigeon/Germany-SH/AI02176/2020 | LSH | FLI | - |
| EPI5057963 | Germany | 2020-11-01 | A/barnacle goose/Germany-SH/AI02180/2020 | LSH | FLI | - |
| EPI813979 | Ireland | 2020-11-01 | A/peregrine falcon/Ireland/20VIR7872-1/2020 | CVRL | IZSVe | Flynn, O., Connaghan, E. *et al.* |
| EPI5058855 | Germany | 2020-11-02 | A/wild goose/Germany-SH/AI02194/2020 | LSH | FLI | - |
| EPI5058432 |  | 2020-11-02 | A/barnacle goose/Germany-SH/AI02190/2020 | LSH | FLI | - |
| EPI5061390 | Germany | 2020-11-03 | A/greylag goose/Germany-SH/AI02207/2020 | LSH | FLI | - |
| EPI5060031 | Germany | 2020-11-03 | A/barnacle goose/Germany-SH/AI02199/2020 | LSH | FLI | - |
| EPI1882989 | Denmark | 2020-11-04 | A/black-headed gull/Denmark/14139-4/2020 | SSI | SSI | Liang, Hjulsager |
| EPI5063706 | Germany | 2020-11-06 | A/barnacle goose/Germany-SH/AI02379/2020 | LSH | FLI | - |
| EPI1848902 | UK | 2020-11-08 | A/brent goose/England/233339/2020 | APHA | APHA | - |
| EPI654958 | Italy | 2020-11-14 | A/mallard/Italy/20VIR7139-73/2020 | IZSVe | IZSVe | Zecchin, B., Fusaro, A. *et al.* |
| EPI710512 | UK | 2020-11-18 | A/whistling duck/England/035643/2020 | APHA | APHA | - |
| EPI5061890 | Germany | 2020-11-18 | A/domestic goose/Germany-SH/AI02884/2020 | LSH | FLI | - |
| EPI1842025 | Poland | 2020-12-03 | A/chicken/Poland/474/2020 | PIWet-PIB | PIWet-PIB | Swieton, Smietanka |
| EPI1916410 | UK | 2020-12-08 | A/red fox/England/AVP-M1-21-01/2020 | APHA | APHA | - |
| EPI1916429 | UK | 2020-12-08 | A/seal/England/AVP-031141/2020 | APHA | APHA | - |
| EPI1846961 | Russian Fed. | 2020-12-12 | A/Astrakhan/3212/2020 | Rospotrebnadzor | VECTOR | - |
| EPI1039231 | Russian Fed. | 2020-12-12 | A/chicken/Astrakhan/321-01/2020 | VECTOR | VECTOR | - |
| EPI1842053 | Poland | 2020-12-16 | A/swan/Poland/MB141/2020 | PIWet-PIB | PIWet-PIB | Swieton; Smietanka |
| EPI1123351 | UK | 2020-12-17 | A/duck/England/043628/2020 | APHA | APHA | - |
| EPI1860071 | Spain | 2021-01-27 | A/anser anser/Spain/297-1_21VIR1230-5/2021 | IZSVe | IZSVe | R. Ramos, S. Zecchin *et al*. |
| EPI1858268 | Austria | 2021-02-01 | A/mute swan/Austria/VIR1085-8/2021 | IZSVe | IZSVe | Wodak, R. Fernandez *et al*. |
| EPI1855975 | Latvia | 2021-02-04 | A/wigeon/Latvia/23903/2021 | BIOR | BIOR | Kibilds, Cvetkova. |
| EPI1858252 | Austria | 2021-02-04 | A/mute swan/Austria/VIR1085-2/2021 | IZSVe | IZSVe | Wodak, R. Fernandez *et al*. |
| EPI1858582 | Lithuania | 2021-02-26 | A/swan/Lithuania/VIR2606-3/2021 | IZSVe | IZSVe | Pridotkas, Jurgelevicius *et al*. |
| EPI1850128 | Czech Rep. | 2021-01-24 | A/mute swan/Czech Republic/1656-1/2021 | SVI Prague | SVI Prague | Nagy, Cernikova, Stara |
| EPI5099463 | Germany | 2021-02-26 | A/chicken/Germany-MV/AI01794/2021 | LALLF | FLI | - |
| EPI5115616 | Germany | 2021-03-04 | A/domestic goose/Germany-SH/AI02100/2021 | LSH | FLI | - |
| EPI5115967 | Germany | 2021-03-05 | A/chicken/Germany-SH/AI02312/2021 | LSH | FLI | - |
| EPI5116326 | Germany | 2021-03-08 | A/chicken/Germany-MV/AI02376/2021 | LALLF | FLI | - |
| EPI5143300 | Germany | 2021-03-10 | A/domestic goose/Germany-MV/AI02558/2021 | LALLF | FLI | - |
| EPI4804850 | Germany | 2021-08-15 | A/seal/Germany-SH/AI05373/2021 | TiHo | FLI | - |
| EPI4805852 | Germany | 2021-08-18 | A/seal/Germany-SH/AI05377/2021 | TiHo | FLI | - |
| EPI4805936 | Germany | 2021-08-19 | A/seal/Germany-SH/AI05379/2021 | TiHo | FLI | - |

APHA, Animal and Plant Health Agency, UK; ARRIAH, Federal Centre for Animal Health, OIE Regional Reference Laboratory, Russian Federation; BIOR, Institute of Food Safety, Animal Health and Environment, Latvia; CVL, Central Veterinary Laboratory, Ministry of Agriculture, Baghdad, Iraq; CVRL, Central Veterinary Research Laboratory, Ireland; FLI, Friedrich-Loeffler-Institut, Germany; IZSVe, Istituto Zooprofilattico Sperimentale delle Venezie, EU/OIE Reference Laboratory and FAO Reference Centre for AI and ND, Italy; LALLF, Landesamt für Landwirtschaft, Lebensmittelsicherheit und Fischerei; LSH, Landeslabor Schleswig-Holstein, Germany; NCB, National Center of Biotechnology, Kazakhstan; NRTSV, National Veterinary Reference Center, Kazakhstan; SVI Prague, State Veterinary Institute Prague, Czech Republic; PIWet-PIB, National Veterinary Research Institut, Poland; Rospotrebnadzor, Center of Hygiene and Epidemiology in Astrakhan Region, Russian Federation; Skóra Station, Prof. Krzysztof Skóra Marine Station, University of Gdańsk, Poland; SSI, Statens Serum Institute, Denmark; TiHo, Stiftung Tierärztliche Hochschule Hannover, Germany; VECTOR, State Research Center of Virology and Biotechnology, Russian Federation.
