# Supplementary material for: Infections with highly pathogenic avian influenza A virus (HPAIV) H5N8 in harbor seals at the German North Sea coast, 2021
Source: Emerg Microbes Infect. 2022 Mar 1;11(1):725–9. doi: 10.1080/22221751.2022.2043726 (PMC8890524; doi:10.1080/22221751.2022.2043726)
Supplement: Supplemental Material [file TEMI_A_2043726_SM9735.zip › Suppl files/Suppl-Table_S3_clean.docx]

Supplementary Table S3. Pathohistological and immunohistochemical findings of lung and brain tissue of investigated harbor seals.

| **harbor seal** | **Organs** | **Histopathology** | **immunohistochemistry  (influenza A nucleoprotein)** |
| --- | --- | --- | --- |
| Meldorf-1 | brain (cerebrum, cerebellum) | mild to moderate^1^, lymphohistiocytic meningoencephalitis with few neutrophils and hemorrhage; single cell necrosis of glial cells and neurons; moderate, multifocal, lymphohistio-cytic vasculitis | multifocal, marked immuno-reactivity within cytoplasm and nuclei of neurons and glial cells |
|  | lung | severe, diffuse, acute hyperemia and alveolar emphysema and edema | negative |
|  | spleen, lymph node, trachea | no significant microscopic findings | not tested |
| Sylt-1 | brain (cerebrum, cerebellum) | moderate, lymphohistiocytic menin-goencephalitis with few neutrophils; single cell necrosis of glial cells and neurons; mild to moderate, multi-focal, lymphohistiocytic vasculitis | multifocal, weak immuno-reactivity within cytoplasm and nuclei of neurons and glial cells |
|  | lung | moderate, diffuse, acute hyperemia;  alveolar emphysema and edema | negative |
|  | spleen, lymph node, trachea | no significant microscopic findings | not tested |
| Sylt-2 | brain (cerebrum, cerebellum) | mild to moderate, lympho-histiocytic meningo-encephalitis with few neutrophils; single cell necrosis of glial cells and neurons; mild to moderate, multifocal, lymphohistio-cytic vasculitis | negative |
|  | lung | mild, diffuse, acute hyperemia and alveolar emphysema and edema and few nematode larvae; mild to moderate multifocal lymphohistio-cytic infiltrates with single cell necrosis | negative |
|  | spleen,  lymph node | no significant microscopic findings | not tested |

^1^ Criteria for determining the severity of lesions is the number of inflammatory cells per high-power field (HPF). Inflammation was categorized as moderate (≤10 cells/HPF), as mild (10-20 cells/ HPF) or severe (≥21 cells/HPF).
