# Supplementary figures and images for: Infections with highly pathogenic avian influenza A virus (HPAIV) H5N8 in harbor seals at the German North Sea coast, 2021
Source: Emerg Microbes Infect. 2022 Mar 1;11(1):725–9. doi: 10.1080/22221751.2022.2043726 (PMC8890524; doi:10.1080/22221751.2022.2043726)

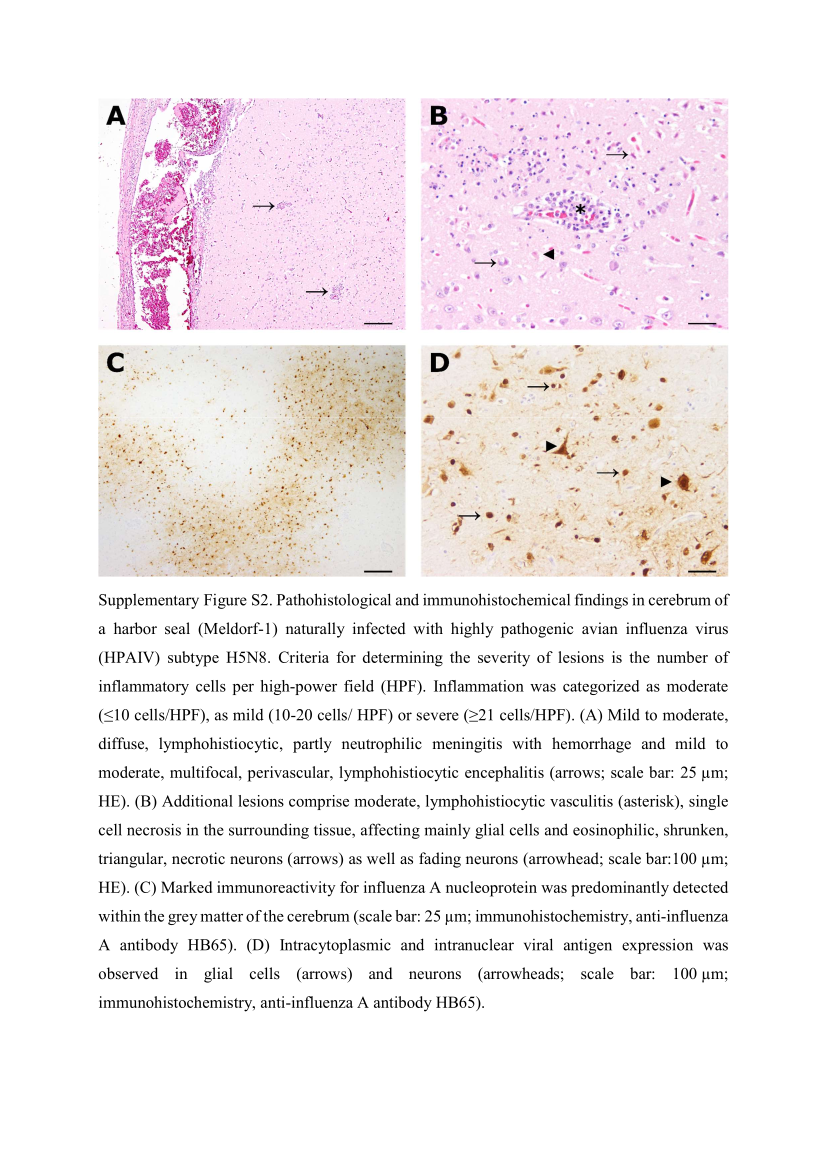

Supplement: Supplemental Material [file TEMI_A_2043726_SM9735.zip › Suppl files/_Suppl-Fig_S2.tif]
